# Factors influencing teachers’ satisfaction and performance with online teaching in universities during the COVID-19

**DOI:** 10.3389/fpsyg.2023.1120662

**Published:** 2023-03-31

**Authors:** Wenbin Du, Ruoyu Liang, Jing Zhang, Lei Wang

**Affiliations:** ^1^School of Humanities, Jiangnan University, Wuxi, China; ^2^School of Design, Jiangnan University, Wuxi, China; ^3^Tianjin Ren’ai College, Tianjin, China; ^4^Department of Mechanical Engineering, Tianjin University, Tianjin, China

**Keywords:** online teaching, performance, satisfaction, expectation confirmation theory, computer self-efficacy

## Abstract

The COVID-19 pandemic has significantly changed the teaching model, promoting educational institutions to initiate more explorations in online teaching. This study examines the factors influencing teachers’ online teaching performance and satisfaction in universities during the COVID-19. We applied a model of technology acceptance (TAM), expectation confirmation (ECM), and computer self-efficacy (CSE) to develop a questionnaire. The survey was used to collect data from 347 teachers from 6 universities in eastern China to identify factors affecting teachers’ performance and satisfaction during the COVID-19. The results indicated that teachers’ performance of online teaching is significantly affected by satisfaction, perceived usefulness, and perceived ease of use of online teaching. Meanwhile, confirmation of online teaching expectations and computer self-efficacy significantly impacted teachers’ satisfaction with online teaching. This work is an original empirical study guided by multiple theories. It contributes to the online education literature and provides advice regarding how teachers’ online teaching satisfaction and performance can be developed in a situation like the one that occurred with COVID-19. This work also broadens the application of TAM and provides an alternative theoretical framework for future research on teachers’ online teaching performance.

## 1. Introduction

Online teaching has become an essential tool in education because of the rapid development of the Internet and information technology (Sepulveda-Escobar and Morrison, 2020). Numerous studies (Yang et al., 2018; Heinrich et al., 2019) have confirmed that online teaching has made a significant contribution to educational opportunities and promoted educational equity (Yang et al., 2018). Empirical research (Mandasari, 2020; Supriyatno et al., 2020; Gopal et al., 2021) has confirmed that online teaching and hybrid teaching (online-offline teaching mode) have positive effects on improving students’ performance, motivation, and learning ability compared with traditional teaching methods.

The large-scale outbreak of the COVID-19 pandemic poses great challenges for face-to-face teaching, and many colleges and universities have closed their campuses and suspended physical teaching and learning activities (Ye et al., 2022). Countries with sophisticated network infrastructure setups have switched to online teaching and have rapidly set up online learning administration systems to bring children back to the classroom. This has been an effective measure to address the shortcomings of face-to-face education during the pandemic (Kruse et al., 2022). For example, the Chinese government mandated distance learning programs for students during school closures because schools experienced huge disruptions in education in the spring of 2020. The policy is named “Classes Suspended, Learning Continues.” Compared with primary and secondary schools, colleges and universities have enforced this policy more strictly. Universities have a good technical platform and resources to support online teaching because they have conducted online courses for decades. According to statistics from the Chinese Ministry of Education, 1.03 million teachers from 1,454 universities delivered online courses to 17.75 million college students to alleviate problems associated with COVID-19 restrictions and face-to-face instruction (Ministry of Education of the People’s Republic of China, 2019).

However, unlike in the past when teachers and students were fully prepared for the online course, the COVID-19 pandemic abruptly forced teachers and students to switch from traditional classroom learning to online learning. Many teachers and students did not have plans and experience in this mode of teaching (Orlov et al., 2021). Thus, a sudden switch from face-to-face teaching to distance learning brings many challenges and constraints (Carrillo and Flores, 2020). With the “rush” and “involuntary” learning, the online learning experience for students and teachers is different from traditional face-to-face learning. In this context, researchers (Gopal et al., 2021; Khan et al., 2021; Ye et al., 2022) have started exploring the teachers’ and students’ satisfaction with online teaching. The goal is to offer suggestions about the quality of online education and the feelings of college teachers and students regarding online learning. Using theories such as online learning ability, connectivism, and achievement goal theory (AGT), researchers have discussed critical success factors that influence the effectiveness of online learning during the pandemic. Gopal et al. (2021) used AGT to identify four independent factors affecting students’ online learning performance, which included teacher quality, curriculum design, timely feedback, and student expectations. Khan et al. (2021) investigated the effects of Internet quality, prior knowledge of information communication technology (ICT), household income, and mother’s education level using quantitative survey methods.

Studies reported that teacher satisfaction, as one of the five pillars of the Sloan Alliance’s high-quality online education, is a key factor that affects the quality of online teaching (Bolliger, 2004; Bolliger et al., 2014). Other studies (Panisoara et al., 2020; Sims and Baker, 2021; Almulla, 2022) have also analyzed the factors influencing teachers’ online teaching satisfaction during the COVID-19 pandemic based on the technology acceptance model (TAM), expectation confirmation theory, and self-efficacy theory. For example, Sims and Baker (2021) surveyed 183 faculties at master’s-level in different Midwest universities to evaluate the rapid transition from traditional courses to online media during the spring 2020 semester. They found that teacher satisfaction with online teaching was low due to a drop in student engagement. Using the TAM, Almulla (2022) also explored the critical factors that influenced online teaching skills, competencies, and the use of digital tools in higher education. The outcome of their survey shows that perceived ease of use and perceived usefulness of using digital devices have a positive impact on lecturers’ behavioral intention to use digital tools during the COVID-19 pandemic.

However, the aforementioned reviewed studies have examined the various factors affecting teachers’ online teaching satisfaction, none of these studies has explained the direct relationship between teachers’ online teaching satisfaction and performance. Satisfaction is an inner psychological experience, which induces changes in performance (Luqman et al., 2017). Blundell et al. (2020) revealed that faculty satisfaction with online teaching during the COVID-19 pandemic was influenced by three main factors: instructor-student interaction, technology’s role, and institutional support. However, they did not explore the relationship between teacher satisfaction and teacher performance.

School closures brought about by the COVID-19 pandemic leave teachers unprepared and unready for online teaching. In China, college teachers have repeatedly used hybrid teaching since 2020, which has brought a lot of teaching pressure on teachers. Therefore, evaluating the factors that influence teachers’ online teaching satisfaction and performance is critical because it has important implications for teachers, education administrators, and technology providers.

This study develops a theoretical model, which integrates the TAM, expectation confirmation theory (ECT), and computer self-efficacy (CSE) to explore the factors influencing the satisfaction and performance of teachers for online teaching behavior. This framework addresses the relative relationship between people’s pre-expectation of a product or service and the actual feeling, based on three levels: the sense of use, the effect of use, and difficulties encountered in the process of use. By proposing and validating the framework, we aim to answer the following research questions:

1. Does teachers’ satisfaction with online teaching affect their online teaching performance?
2. Which elements are critical drivers for teachers’ performance and satisfaction in online teaching?
3. To what extent do these drivers explain teachers’ online teaching satisfaction?

We review past literature and conduct an empirical analysis to answer these questions.

The remainder of the paper is organized as follows. Section “Literature review” reviews the existing studies that use TAM, ECT, and CSE in explaining teachers’ satisfaction and performance with online teaching. Section “Research model and hypotheses” proposes the research model and hypotheses, and Section “Research methodology” describes research methods, including structural measurements and data collection. Section “Results” presents data analysis and hypothesis testing results, and Section “Discussion” discusses the results. Finally, Section “Conclusion” concludes the paper.

## 2. Literature review

### 2.1. Performance

Job performance can be defined as the total expected value to the organization of the discrete behavioral episodes that an individual conducts over a standard period of time (Wen et al., 2019). Teaching performance refers to a teacher’s work shown in carrying out the tasks, roles and responsibilities in order to achieve school goals (Kusumaningrum et al., 2019). Teaching performance is the most significant contribution to the educational process. It determines whether teachers can teach and deliver reliable teaching results in new teaching environments and when facing new demands and challenges. With an increasing demand for online teaching, teachers’ performance has become a critical factor in evaluating online education quality. Zhu et al. (2022) pointed out that online teaching performance was affected by teachers’ concepts, abilities, and experience, especially when applying ICT, which were essential factors affecting the effectiveness of online teaching. Xiang et al. (2021) confirmed that teachers’ performance in online teaching, such as having teaching proficiency, having teaching information, and answering questions, has significantly affected the quality of online teaching.

### 2.2. Satisfaction

Satisfaction refers to the post-consumption experience and post-use decidsion of a product, system, and service (Ali et al., 2021). Teachers’ satisfaction is an essential factor in evaluating the quality of online courses and has been extensively analyzed in research focusing on modern educational technology-driven instruction (Blundell et al., 2020). Wingo et al. (2017) summarized 67 empirical studies about online teaching using TAM 2 framework. The results show that factors affecting teachers’ online teaching satisfaction are barriers to student success in online classes, technical support needs, uncertainty about the image of online instructors, desire for a reasonable workload, and class enrollments in online courses.

### 2.3. Technology acceptance model

TAM is first proposed by Davis (1985), and it has become a classic model to predict and explain people’s attitudes and intentions to use new information technologies or information systems. The field of education is no exception (Scherer et al., 2019; Han and Sa, 2021). TAM assumes that individuals’ behavioral preferences to use IT depend on their satisfaction and perceived ease-of-use (refers to the ease of using a specific type of IT) and perceived usefulness (refers to the extent to which the technology improves work performance). These are the two most essential factors influencing individuals’ attitudes toward usage (Venkatesh and Davis, 2000). With the increasing application of new ICT in education (Akram et al., 2021; Bajaj et al., 2021), the effectiveness of TAM has been proved by empirical research to explain teachers’ attitudes toward online teaching (Siyam, 2019; Mailizar et al., 2021). For example, Bajaj et al. (2021) used TAM to explain teachers’ intention to continue using online teaching tools during COVID-19. They found that ease of use of technology positively influences teachers’ attitudes toward online teaching. Compared with female teachers, male teachers are more inclined to use online teaching platforms during COVID-19. Akram et al. (2021) used empirical analysis to demonstrate that Pakistani teachers faced challenges in online teaching during the COVID-19 pandemic. They found a favorable attitude of faculty members toward using virtual education platforms. However, faculty members encountered several challenges that caused restrictions in accomplishing competent teaching and learning. For example, they lacked experience in conducting online classes and were not given adequate technical assistance or ICT infrastructures. The above-reviewed studies in this subsection have shown that TAM can provide clear variables for identifying factors influencing teachers’ satisfaction with online teaching and for offering considerable operability and effectiveness in examining the attitude toward online teaching. Given the above context, this study uses TAM to assess Chinese university teachers’ satisfaction with online teaching during the COVID-19 pandemic.

TAM estimates technology intention through subjective perceptions of the “usefulness” and “ease of use” of the technology. However, perceived usefulness and ease of use are broad constructs and cannot capture the specific instrumentality of users’ technology adoption behavior. Therefore, studies (Guo et al., 2017; Du et al., 2022) often extend and adopt the TAM in analyzing users’ technology intention for examining the specific factors influencing user experience.

### 2.4. Expectation–confirmation model

Expectation–Confirmation Model (ECM), sourced from the expectation–confirmation theory (ECT; Oliver, 1980), is the most adopted model used to analyze the satisfaction development process. The model reveals the correlation between satisfaction and consumers’ post-adoption expectations of the service/product based on their experience (Nam et al., 2020). However, Bhattacherjee (2001) argues that the theory has some weaknesses because it ignores potential changes that customers expect after a consumption experience and the impact of those changes on subsequent cognitive processes. In this respect, Bhattacherjee developed an ECM of information system (IS) continuance to explore users’ satisfaction and continuance behaviors in the context of ISs. This model modifies the ECT by extending the post-acceptance expectation of the user and expressing it in terms of perceived usefulness.

Recently, ECM of IS continuance has been extended into e-learning to explore learner satisfaction for online courses. Daneji et al. (2019) explained examines the effects of students’ perceived usefulness, confirmation and satisfaction on MOOC continuance intention by ECM and revealed that students’ confirmation has significant effects on their perceived usefulness and satisfaction. Zhu et al. (2020) examined the changes in university students’ attitudes toward online learning by ECM and TTF, and found that university students’ online learning attitudes were generally positive. Their continuous intention to learn online was significantly predicted by their self-regulated learning capability, online interactions, attitudes, and online learning intention. They verified that expectation confirmation was a significant predictor for gaining perceived usefulness.

### 2.5. Computer self-efficacy

CSE represents an individual’s efficacy in performing specific computer-related tasks within the domain of general computing (Teo et al., 2018). As an application of self-efficacy in electronic information, CSE is derived from the social-cognitive theory of individual behavior, which explains human behavior in terms of the interplay between behavioral, cognitive, and environmental determinants (Bandura, 1997). Compeau and Higgins (1995) applied the concept of self-efficacy to information technology, which referred to an individual’s ability to accomplish a particular task using a computer system (Shen and Eder, 2009). The CSE model has focused on specific applications from a broad range of general computing tasks. In the e-learning field, several studies on online learning satisfaction highlight the importance of the CSE model for a successful online learning experience. Wang et al. (2019) found that CSE significantly affect the intention of students to continue using Cloud e-learning application. Likewise, Katsarou (2021) also believed that high CSE levels were important factors that promoted students’ academic success in the current digital era because CSE levels reduced technology anxiety and increased positive attitudes toward e-learning.

The CSE model is appropriate for this study because of two reasons: First, CSE provides new insights into computer system use behavior, which proves that individuals with a strong sense of capability are more willing to accept and use the system (Jan, 2015; Abdullah and Mustafa, 2019). Second, the model provides a concise and structural way of examining the impact of a user’s inherent ability awareness on the user’s experience in technology. In the urgent shift to online teaching caused by COVID-19, teachers with high technology awareness are more active in overcoming unexpected online teaching challenges, generating a better online teaching technology experience, and achieving better user satisfaction (Zhao and Zhao, 2021). Therefore, we apply this model to online teaching to identify such teachers’ behaviors and the consequences.

## 3. Research model and hypotheses

**Figure 1:**
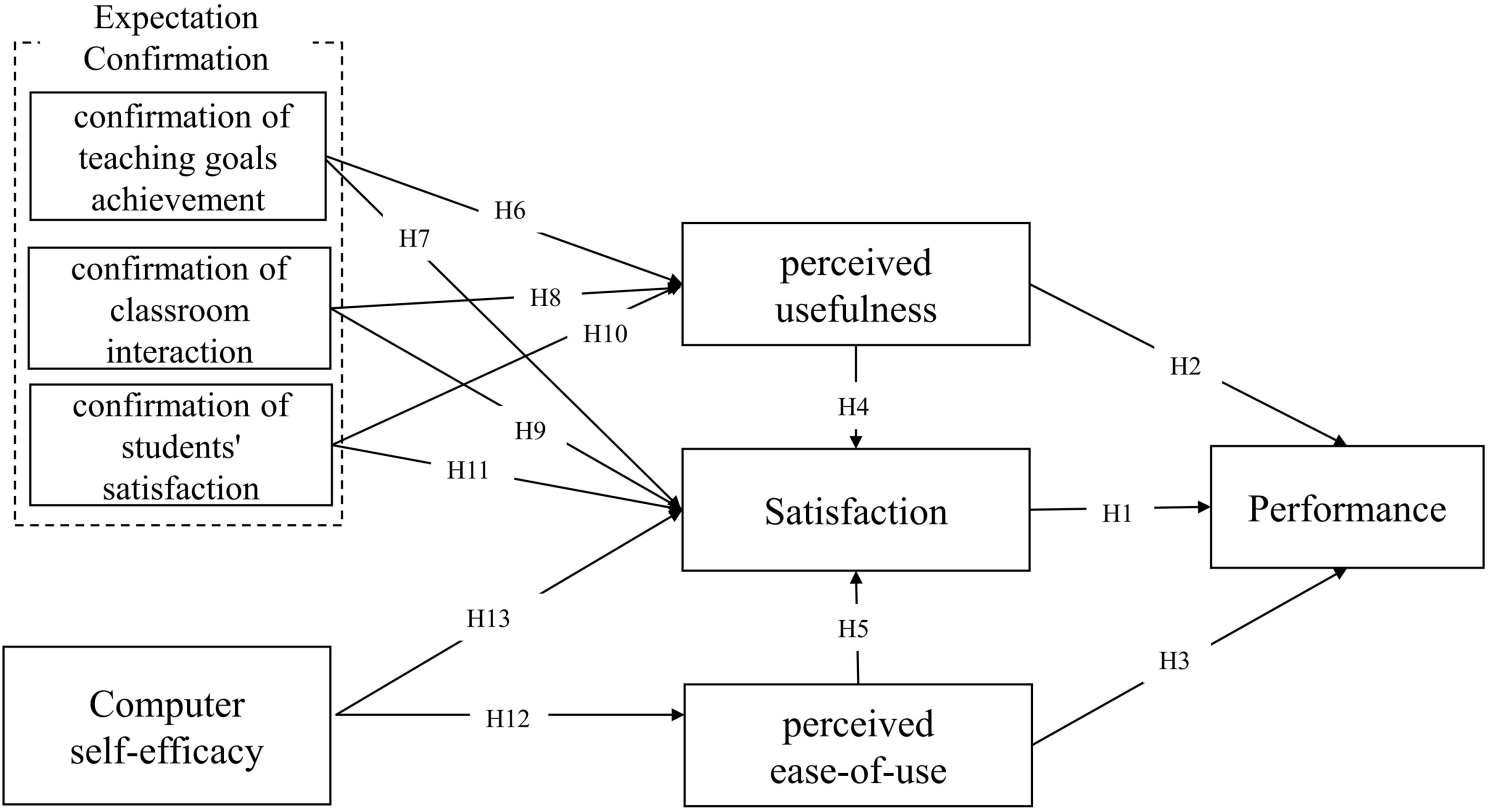
Research model.

The study established an integrated model of TAM, ECM, and CSE to explore the predictors of teachers’ satisfaction in the online teaching context. In the research model, expect confirmation factors (i.e., expectations of teaching goal achievement, expectations for a quality classroom interaction, and expectations for student satisfaction) were applied to represent perceived usefulness. We also adopted teachers’ ability in using online teaching platforms and in solving technical difficulties associated with the CSE factors. Figure 1 depicts the research model and the proposed hypothesized relationships.

### 3.1. Relationship between online teaching satisfaction and teaching performance

The relationship between satisfaction and performance has been examined in various organizational settings. Petty et al. (1984) conducted a meta-analysis on the relationship between job satisfaction and job performance from 1963 to 1983. They confirmed a positive relationship between job satisfaction and performance. In the field of education, Torres (2019) describes teachers’ satisfaction as the level of fulfillment gained from work. The critical impact of teachers’ job satisfaction on their teaching performance has been demonstrated by many studies (Li et al., 2018; Wolomasi et al., 2019; Sudirman et al., 2021). For example, Li et al. (2018) found that job satisfaction positively supports teachers’ job performance. Therefore, hypothesis 1 is formulated.

*H1*: Teachers’ satisfaction is positively associated with teachers’ performance in online teaching.

### 3.2. Relationship between perceived ease of use, perceived usefulness, online teaching satisfaction, and performance

Perceived ease of use of online teaching is the degree of adaptability of teachers to the online teaching model and the comfort level of teachers to use online teaching tools. Several studies have confirmed that adopting digital tools for teaching and learning is a critical indicator of attitude toward technology adoption (Briz-Ponce and García-Peñalvo, 2015; Mehta et al., 2019). According to Hu and Xie (2020), Chinese college instructors adopted more than 10 online teaching methods during the pandemic, including live broadcast + online interaction, recording + online interaction, student self-learning + online interaction, SPOC (small private online course), and PPT (PowerPoint presentation) + online discussion. Online interaction is a crucial part of all teaching methods. Different teaching methods are accompanied by various online teaching tools, which significantly challenges teachers’ online teaching ability. Given the existing research on the application of TAM in IT-based teaching, this study considers the perceived ease of use of online instruction, which comprises three components: perceived comfort of operating online teaching tools, perceived ease of online class design, and perceived ease of interaction in online classes.

Perceived usefulness is defined as digital tools used to solve existing pedagogical problems or improve pedagogical effectiveness (Pańkowska et al., 2020). Lin et al. (2011) argue that teachers’ perceived usefulness to online teaching is based on how this teaching mode helps achieve their learning goals. Sims and Baker (2021) explore faculty perceptions of online teaching during COVID-19 based on three aspects: the quality of online teaching, the transition from face-to-face classes to online classes, and students’ performance and course completion. All three are adopted in this study as dimensions of the perceived usefulness of online teaching.

Satisfaction is defined as a sense, derived from the post-assessment of a consumer product or service experience (Liu et al., 2018). When the perceived performance meets or exceeds individual expectations, the feeling of satisfaction occurs (Palacio et al., 2002). Perceived usefulness and ease of use are stable indicators of users’ emotional disposition toward technology than other factors, such as technology safety. Thus, some studies have indicated that teacher readiness and use of the TAM positively and significantly impact teacher performance during COVID-19 (Sulistiyani and Nugroho, 2022). Accordingly, the higher the quality of online teaching to solve the problem of educational interruption caused by the epidemic, the higher the satisfaction with the technology adopted within teachers. The ease of online teaching and tools also affects teachers’ emotions and effectiveness, which in turn affects teachers’ satisfaction and performance. Therefore, the following hypotheses are formulated.

*H2*: Perceived usefulness is positively associated with teachers’ performance in online teaching.

*H3*: Perceived ease of use is positively associated with teachers’ performance in online teaching.

*H4*: Perceived usefulness is positively associated with teachers’ satisfaction with online teaching.

*H5*: Perceived ease of use is positively associated with teachers’ satisfaction with online teaching.

### 3.3. Relationship between expectation conformation, perceived usefulness, and online teaching satisfaction

The confirmation of expectations suggests that users obtain expected benefits through usage experiences, which leads to a positive effect on users’ satisfaction (Bhattacherjee, 2001). Expectation confirmation is used to assess the functionality and usefulness of online teaching pedagogical applications (Tan and Shao, 2015; Zhou, 2017). Empirical studies reveal that this desired conformation-to-satisfaction relationship influences perceived usefulness. Lee (2010) found that users’ confirmation of expectations was positively correlated to their perceived use based on the predicted users’ continuance intention toward e-learning using an expectation–confirmation extension model. Alraimi et al. (2015) found a significant relationship between users’ confirmation of expectations and the perceived usefulness of MOOCs’ continuance.

ECM variables implicitly assume that behavior is voluntary. Thus, it is crucial to explore factors that influence these variables and how they are manipulated to improve teachers’ experience in online teaching. Webster and Hackley (1997) summarized the factors that affected distance learning into four aspects: technical characteristics, teacher characteristics, curriculum characteristics, and learner characteristics. Using confirmation of expectations, Zhou et al. (2017) divided the use of e-learning into three aspects: knowledge outcome, performance proficiency, and social influence. These represent the learners’ perception of subject matter understanding, the degree to which an individual possesses the required knowledge, skills, abilities, and influence from others’ evaluations (Yu et al., 2010; Wei et al., 2011; Wan et al., 2012). Zhu et al. (2022) and Zheng et al. (2022) have shown that in the face of unexpected online teaching, teachers have developed the following adaptive anxiety: teaching effect, teacher-student interaction, and student learning guidance. Therefore, we adopted the perceived usefulness of online teaching and identified teachers’ expectations of teaching goal achievement, online classroom interaction, and student satisfaction.

Teaching achievement is how well teachers complete their teaching objectives through online teaching. Achievement goal theory suggests that motivation and achievement-related behavior can easily be understood through purpose and people’s engagement in activities (Bardach et al., 2020; Urdan and Kaplan, 2020). As a compensatory teaching method during the epidemic, the most crucial purpose of online teaching is to help teachers complete their teaching plans and achieve their teaching goals. Teachers will perceive the usefulness of online teaching and become satisfied when online learning achieves impressive teaching goals. Hence, we hypothesized:

*H6*: Confirmation of teaching goal achievement while using online teaching has a positive effect on perceived usefulness.

*H7*: Confirmation of teaching goal achievement while using online teaching has a positive effect on teachers’ satisfaction.

Classroom interaction refers to the exchange, cooperation, and communication between teachers and students. Teaching is a behavioral interaction process between teachers and students. In online teaching environments, a reduction in social presence and appropriate classroom interaction activities has become a thorny problem for teachers because of time and space constraints. Existing research has found that positive classroom interactions can enhance the student’s learning experience and improve teaching quality (Malik et al., 2010; Martínez-Argüelles and Batalla-Busquets, 2016). Teachers will perceive the usefulness of online teaching and become satisfied when high-quality interactions are established. Hence, we hypothesized::

*H8*: Confirmation of online classroom interaction has a positive effect on perceived usefulness.

*H9*: Confirmation of online classroom interaction has a positive effect on teachers’ satisfaction.

According to Awidi and Paynter (2019), student satisfaction refers to a positive affective state that results from the evaluation of the teaching module and method. In classroom teaching, teachers can captures the affective state of students through dialog with students and observe students’ movements and facial expressions, and to understand students’ learning investment and control teaching progress (Yang and Zhang, 2020). If students show a positive affective state with classroom teaching, teachers will believe that the education has achieved good results. Therefore, we hypothesized:

*H10*: Confirmation of student satisfaction while using online teaching has a positive effect on perceived usefulness.

*H11*: Confirmation of student satisfaction while using online teaching has a positive effect on teachers’ satisfaction.

### 3.4. Relationship between computer self-efficacy, perceived ease of use, and online teaching satisfaction

Studies have shown that CSE influences users’ perceptions of information technology/product use (Jan, 2015). Users with higher CSE generally experience fewer barriers to technology, and this improves their positive attitude toward the use of the technology (Cherry and Flora, 2017; Teo et al., 2018).

Numerous studies have confirmed the positive correlation between CSE and perceived ease of use of technology. Bai et al. (2021) found that CSE significantly enhanced the ease of use of technology, indicating that when teachers believed in their skills to use ICT tools, they were more likely to use specific ICT tools to enhance their teaching performances. Self-efficacy includes an individual’s belief in one’s skills to complete specific tasks, to enhance an individual’s resistance to obstacles, and to enhance persistence in the face of setbacks and determination to complete complex tasks (Bandura, 1997). In the online education environment, the participants’ belief in their ability to use online teaching technology and their confidence in facing difficulties associated with online teaching. Some studies believe in primary manifestations of the participants’ computer self-efficacy (Hampton et al., 2020; Huang et al., 2022). Accordingly, the following hypotheses are proposed:

*H12*: Teachers’ computer self-efficacy for online teaching positively affects perceived ease of use.

*H13*: Teachers’ computer self-efficacy for online teaching positively affects teachers’ satisfaction.

### 3.5. Control variables

Since prior researchers (Guo et al., 2017; Li et al., 2018) considered that participants’ individual demographic characteristics might potentially influence the relationships of performance, we added gender and teaching years as control variables in the analyses.

## 4. Research methodology

### 4.1. Questionnaire design

We developed a questionnaire to analyze the factors influencing teachers’ performance and satisfaction with online teaching. Eight constructs have been measured. Some items on the questionnaire were self-developed based on the research results of previous studies (Compeau and Higgins, 1995; Bhattacherjee, 2001; Pecheone and Chung, 2006; Zhou, 2017; Teo et al., 2018; Hampton et al., 2020; Qin and Zhou, 2020; Wei and Chou, 2020; Bajaj et al., 2021; Sims and Baker, 2021; Sharma and Saini, 2022); whereas others were adapted from measures that previous studies had validated. The Performance Assessment for California Teachers (PACT) was used to evaluate teacher performance in five dimensions: (1) how to plan learning goals and student needs, (2) how to engage in purposeful instruction and reflect on the results, (3) how to evaluate student learning, (4) how to prepare for next steps for individual students and (5) how to prepare for the class. This assessment has been widely accepted to assess in-depth teaching performance (Pecheone and Chung, 2006). Therefore, we adapted the item on teacher performance from the PACT. The scales measuring satisfaction were self-developed based on the research results of Bhattacherjee (2001). The perceived usefulness and ease of use items were adapted from Qin and Zhou (2020) and Sharma and Saini (2022), respectively. For expectation confirmation, the items measuring confirmation for online classroom interaction and student satisfaction were adapted from Hampton et al. (2020) and Bajaj et al. (2021) models. Moreover, the items for confirmation of instructional goal achievement were self-developed based on results by Zhou (2017) and Sims and Baker (2021). The items for computer self-efficacy were adapted from Compeau and Higgins (1995), Wei and Chou (2020), and Teo et al. (2018).

The questionnaire items were written in Chinese using a seven-point Likert scale ranging from 1 (“strongly disagree”) to 7 (“strongly agree”). As some of the items were in English, we translated them into Chinese and made the necessary modifications for easy understanding. These measure items are listed in Table 1.

**Table 1:** Observed variables influencing teachers’ online teaching behavior.

Dimension	Observed variables
Online teaching performance (a)	a1 Be able to make online teaching plans around learning goals and student needs
	a2 Be able to teach online with a purpose
	a3 Be able to reflect on the effectiveness of online teaching
	a4 Be able to provide positive feedback on student learning
	a5 Be able to help students develop personalized learning plans
Online teaching satisfaction (b)	b1 Satisfaction with the teaching effect of online teaching
	b2 Satisfaction with the teaching experience of online teaching
	b3 Feel the values of online teaching
Perceived usefulness (c)	c1 Effectively organize learning resources
	c2 Effectively implement classroom teaching activities
	c3 Effectively carry out classroom teaching evaluation
Perceived ease-of-use (d)	d1 Ease of operating online teaching platforms and tools
	d2 Ease of providing flexible teaching features
	d3 Ease of organizing online classroom teaching
Confirmation of teaching goal achievement (e)	e1 Complete expected teaching progress
	e2 Complete expected teaching goals
	e3 Improve teaching effectiveness.
Confirmation of online classroom interaction (f)	f1 Establish effective communication with students
	f2 Establish effective communication among students
	f3 Catch individual students’ needs.
Confirmation of student satisfaction (g)	g1 Students show satisfaction with the classroom effect
	g2 Students show satisfaction with learning achievement
	g3 Students show satisfaction with classroom interactions
Computer self-efficacy of online teaching (h)	h1 Confidently perform essential functions of an online teaching platform
	h2 Confidence to carry out online teaching or online and offline hybrid teaching.
	h3 Could complete online teaching if used similar technologies.
	h4 When have technical difficulties in online teaching, specialized instruction is available

### 4.2. Sample and data collection

Samples have been collected from six universities from eastern China. These universities conducted online teaching several times in 2020–2022 because of the COVID-19 epidemic. Teachers and students have turned to online education at least three times in the past six semesters. For example, these six universities have just launched online teaching in the summer semester of 2022. They also used an online teaching platform for the final exam. Teachers at these schools have rich online teaching experience. Therefore, they are ideal places for our research. We contacted some teachers at six universities in eastern China through former classmates, colleagues, and personal contacts. Relying on these teachers we are familiar with, we randomly recruited 50–60 teachers from each school to participate in the questionnaire survey. Finally, a total of 355 teachers agreed to participate in the survey.

We consulted seven experts in higher education and teachers’ professional development for the questionnaire content validity before the survey. Then, we revised the questionnaire structure and language using experts’ feedback. We launched a pre-survey, distributed 150 questionnaires across two universities in Jiangsu Province, China, and collected 142 results. We tested the data group’s internal consistency reliability using Cronbach’s alpha coefficient. From the results, Cronbach’s alpha coefficient of the questionnaire was 0.836; Cronbach’s alpha coefficients for each component were 0.943, 0.852, 0.798, 0.819, and 0.813. The questionnaire had good reliability because all these Cronbach’s alpha coefficients were above 0.7. Thereafter, we launched an 8-week survey. We emailed these teachers the link to the survey and a description of the purpose of the study. However, among the returned questionnaires, eight questionnaires showed obvious regular answers. Therefore， we collected 347 valid questionnaires for data analysis. Table 2 shows the demographic characteristics of the respondents.

**Table 2:** The demographic characteristics of the respondents (*N* = 347).

Measure	Items	Frequency	Percentage (%)
Gender	Male	186	53.6
	Female	161	46.4
Teaching years^*^	1–5	187	53.8
	5–10	123	35.4
	more than 10	37	10.8
Teaching Area^**^	Philosophy	35	10.2
	Economics	21	5.9
	Management	32	9.2
	Law	15	4.5
	Education	61	17.5
	Literature	36	10.4
	History	26	7.6
	Science	34	9.8
	Engineering	29	8.4
	Agriculture	0	0
	Medicine	12	3.3
	Military	0	0
	Art	46	13.1

^*^In China, universities usually sign employee contracts with teachers every five years. ^**^According to the Subject Catalog of Degree Conferment and Talent Cultivation, there are 13 disciplines of undergraduate education in China.

### 4.3. Data processing and reliability and validity testing

The study model was analyzed using partial-least-squares (PLS) regression. PLS is a widely used model for the regression of multiple dependent and independent variables and for handling latent variables with multiple indicators in a single model. Meanwhile, It is also suitable for dealing with non-normal distribution data and small sample sizes. Many studies have proved PLS is the preferred approach for theory development, exploratory research, and existing theory extension (Gefen et al., 2000; Hair et al., 2011). This work’s primary research goals are to develop satisfaction and performance theory and explore the factors that influence teachers’ online teaching performance and satisfaction. Therefore, PLS is appropriate for our study. According to Chang et al. (2014), data analysis was performed in two steps: (1) the reliability and validity of the measurement model were analyzed; (2) the data of the structural model were interpreted. Data processing and analysis were performed using the software SmartPLS 3.3 developed by the University of Hamburg (Ringle et al., 2015).

The reliability and validity of all variables were tested to verify the measurement model. From the results in Table 3, the construct reliability of all variables in any construct is greater than 0.7, proving that the data are sufficiently reliable (Gefen et al., 2000).

**Table 3:** CR, AVE, and correlation coefficients of latent variables.

Dimension	CR	AVE	A correlation coefficient of latent variables							
			1	2	3	4	5	6	7	8
1. Online teaching performance	0.77	0.73	0.85							
2. Online teaching satisfaction	0.73	0.79	0.44	0.89						
3. Perceived usefulness	0.82	0.65	0.39	0.55	0.81					
4. Perceived ease-of-use	0.81	0.82	0.12	0.58	0.61	0.91				
5. Confirmation of teaching goal achievement	0.92	0.76	0.52	0.43	0.38	0.57	0.87			
6. Confirmation of online classroom interaction	0.74	0.69	0.41	0.22	0.33	0.56	0.62	0.83		
7. Confirmation of student satisfaction	0.85	0.61	0.27	0.31	0.26	0.42	0.29	0.47	0.78	
8. Computer self-efficacy of online teaching	0.89	0.64	0.69	0.39	0.48	0.30	0.23	0.51	0.66	0.80

The numbers highlighted in gray are the square roots of AVE and should be greater than other correlation coefficients.

According to Fornell and Larcker (1981), the convergent validity of the measurement model is ascertained when all observed variable loadings are greater than 0.7, and the average variance extracted (AVE) of all variables is greater than 0.5. Table 4 shows that the loadings of all observed variables are greater than 0.7, and the AVE is between 0.61 and 0.82, proving that the convergent validity of the observation model meets the statistical requirements.

**Table 4:** Results of confirmatory factor analysis.

	Average	Standard deviation	Loading
a1	4.95	0.83	0.81
a2	5.23	1.04	0.85
a3	4.52	0.80	0.89
a4	4.87	0.94	0.90
a5	4.30	0.88	0.82
b1	4.41	1.12	0.86
b2	4.52	0.95	0.85
b3	5.01	0.99	0.80
c1	4.66	0.82	0.92
c2	4.78	0.85	0.88
c3	4.36	0.97	0.82
d1	5.19	0.83	0.91
d2	4.84	0.81	0.88
d3	4.27	1.09	0.85
e1	5.36	0.82	0.87
e2	4.80	0.92	0.83
e3	4.35	0.96	0.84
f1	4.96	0.89	0.84
f2	4.43	1.01	0.91
f3	3.89	1.17	0.85
g1	4.22	0.98	0.83
g2	3.87	0.93	0.87
g3	4.18	0.86	0.95
h1	4.81	1.08	0.82
h2	4.60	0.97	0.93
h3	5.07	0.83	0.87
h4	4.56	1.03	0.89

Additionally, discriminant validity must satisfy the following two criteria: the correlation coefficient between variables should be less than 0.85, and the square root of the AVE of each variable should be greater than the correlation coefficients of other variables in the model (Fornell and Larcker, 1981). Table 3 shows that the correlations between variables and the square roots of AVE are found on the diagonal of the table, all satisfying the above two criteria and proving that the measurement model has acceptable discriminant validity.

The variance inflation factor (VIF) was calculated for all variables to prevent multicollinearity problems. According to Hair et al. (2006), a model suffers from multicollinearity problems when the VIF exceeds 10. We ran VIF tests, and the calculation result showed that none of the VIF values exceeded 5.1, confirming the absence of significant multicollinearity problems in the model.

Furthermore, common method bias (CMB) should be tested as all samples were collected simultaneously with self-report measures. According to Podsakoff and Organ (1986), Harmon’s one-factor test was recommended to assess CMB. The result showed that the first factor accounted for only 31.6% of the overall variance, indicating that no single factor could significantly influence the overall variance of the model. In addition, according to Pavlou (2005), the CMB will affect the data validity when the correlation coefficient among constructs exceeds 0.9. Table 3 shows that the correlation coefficient between any two latent variables is less than 0.9, suggesting that CMB is not a significant problem for this work.

## 5. Results

**Figure 2:**
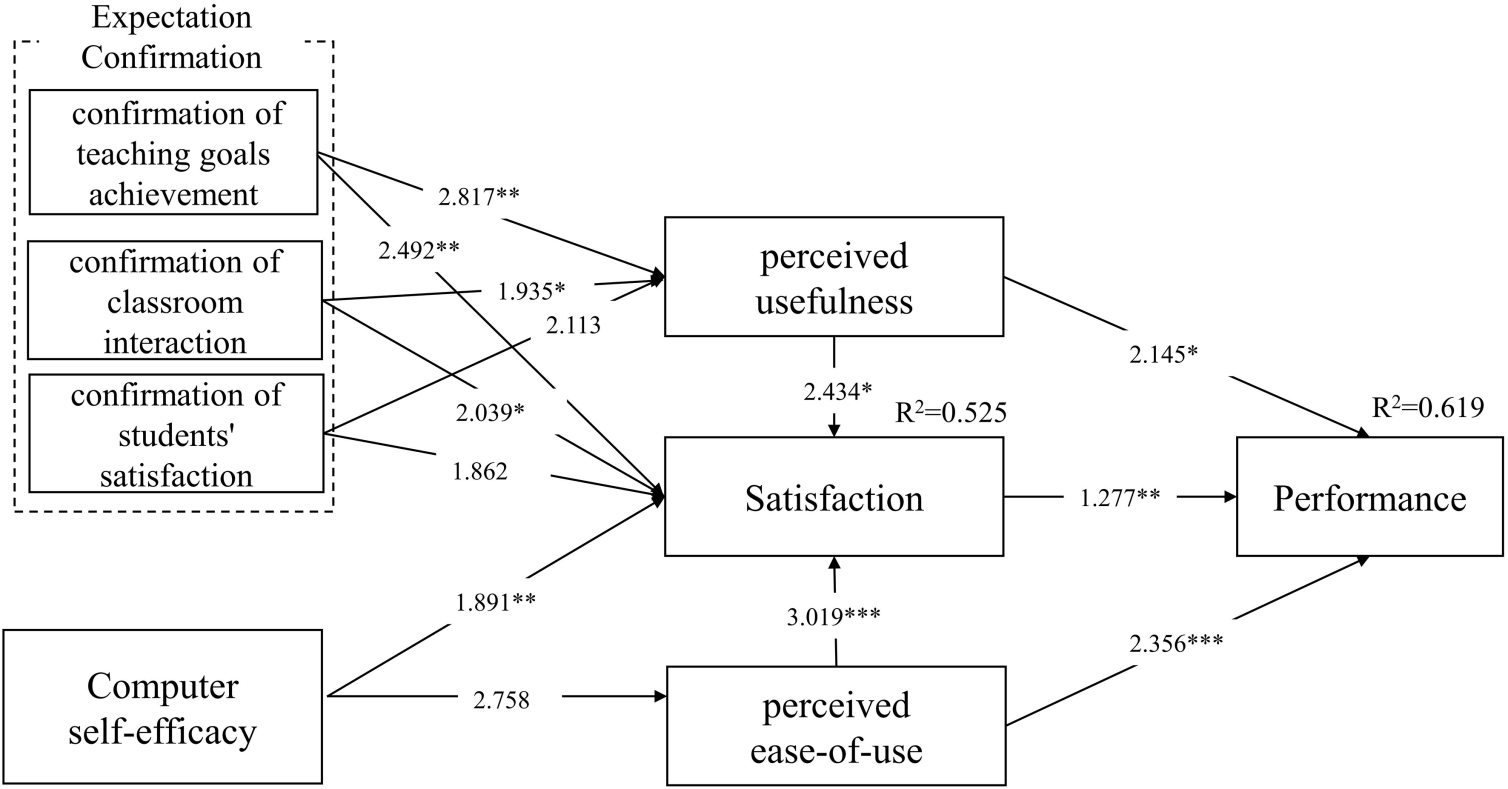
Structural equation modeling results. ^*^p < 0.05. ^**^p < 0.01. ^***^p < 0.001.

We adopted a bootstrap method to analyze the significance of each variable path and the structural equation’s explanatory power. The goal is to gain insights into the effect of each potential factor on teachers’ satisfaction and performance in online teaching. The bootstrap method used a repeated sampling of valid sample data for statistical analysis, with a sample size of 500. The structural equation modeling results are shown in Figure 2.

### 5.1. Teachers’ performance and attitude toward online teaching

The results showed that teachers’ online teaching satisfaction significantly affected their performance during the COVID-19 pandemic, suggesting that hypothesis 1 is supported.

The regression model revealed that teachers’ perceived usefulness and ease of use of online teaching significantly had positive effects on their online teaching performance, which supported hypotheses 2 and 3. Additionally, the contribution of perceived ease of use of online teaching to teachers’ performance was more significant than that of perceived usefulness.

The regression model has shown that the teachers’ perceived ease of use and usefulness in online teaching significantly has a positive effect on their online teaching satisfaction. Hypotheses 4 and 5 have been supported. Additionally, the contribution of perceived ease of use to teachers’ satisfaction was more significant than that of perceived usefulness.

### 5.2. Expectation confirmation and online teaching satisfaction

Regarding expectation confirmation, the regression model analysis revealed that confirmation of teachers’ expectations for the teaching goals and classroom interaction had a significant effect on online teaching’s perceived usefulness. These results supported hypotheses 6, and 8.

The effect of confirmation of students’ satisfaction on perceived usefulness was not significant; thus, hypothesis 10 was not supported.

Furthermore, teachers’ expectations confirmation of the achievement of teaching goals and classroom interaction significantly affects teaching satisfaction, which supported hypotheses 7, 9.

The effect of expectation confirmation of students’ satisfaction on teachers’ online teaching satisfaction was not significant; thus, hypothesis 11 was not supported.

### 5.3. CSE and online teaching satisfaction

Based on the CSE factor analysis, the regression model showed that the effect of teachers’ CSE on perceived ease of use was not significant. Thus, hypothesis 12 was not supported. However, CSE had a significant effect on teachers’ online teaching satisfaction, which supported hypothesis 13. Additionally, we measured the explanatory power of the model using R-Squared (R^2^). The R^2^ for teachers’ performance and teachers’ satisfaction were 0.0619 and 0.525, respectively, showing that performance and satisfaction had 61.9 and 52.5% of the variance in teachers’ online teaching behavior. Thus, the research model explained most of the variance in teachers’ performance in online teaching, revealing that the model had strong explanatory power.

### 5.4. Evaluating control variables

one-way ANOVA has been applied to examine the effects of control variables (i.e., gender and teaching year) on teachers’ online teaching performance (Panigrahi et al., 2021), and the results revealed that there were no significant differences in teaching performance in different gender and teaching year groups (both *p* > 0.1). These findings suggest that the effects of the control variables are insignificant.

## 6. Discussion

### 6.1. Major findings

This study uses a model that integrates TAM with ECM and CSE to analyze the influencing mechanism of online teaching satisfaction and performance of Chinese university teachers during the COVID-19 epidemic. We obtained some findings after the regression analysis.

First, the empirical results confirm that teacher satisfaction, teachers’ perceived usefulness to online teaching, and perceived ease of use are positively correlated with teachers’ online teaching performance. Typically, job satisfaction affects job performance, implying a relationship between morale and productivity. Higher morale improves productivity (Strauss, 1968). Thus, studies believe that a positive assessment of an attitude object predisposes people to engage in positive behaviors (Dewaele, 2019; Donehower Paul et al., 2020). Conversely, an unfavorable evaluation can predispose an individual to hinder or oppose this behavior (Eagly and Chaiken, 1993; Dunn et al., 2018). Judge et al. (2001) conducted meta-analytic reviews of 1,008 studies on the relationship between job satisfaction and job performance; they found that the mean accurate correlation of these two factors was moderate (0.30), which suggests that satisfaction may not exert effects on job performance. Fortunately, our empirical results demonstrate that a correlation exists in online teaching. Teachers’ online teaching satisfaction has a significant impact on teachers’ online teaching performance. Therefore, the quality of online education is vital, and it is pivotal to adopt measures to improve teachers’ online teaching satisfaction.

Second, our results confirmed a positive correlation between teachers’ perceived ease of use, perceived usefulness, and teacher performance in online teaching. Perceived ease of use has a more significant impact on teacher performance. This result is consistent with the results presented by Zhu et al. (2022). They found that teachers who demonstrated high performance in online teaching had higher adaptability to online teaching and a higher ability in using online teaching tools. This adaptability and knowledge can make teachers feel confident and calm about online teaching. Additionally, a rich online teaching experience can help teachers organize online teaching effectively and enhance online teaching performance. As we presume, teachers can improve their online teaching performance and guarantee the quality of online teaching if they receive trusted teaching support and technical support.

Our research also shows that teachers’ perceived usefulness and ease of use in online teaching positively influence teachers’ satisfaction with online teaching. This result is consistent with the findings presented by previous studies regarding teachers’ online teaching satisfaction (Tan and Shao, 2015; Bajaj et al., 2021).

Additionally, the findings underscore the critical role of perceived ease of use in achieving teacher satisfaction, revealing teachers’ convenience in online teaching may arouse their intention. This strategy has more weight than making teachers understand the value of online teaching and students’ academic achievement. Sharma and Saini (2022) also found that teachers’ perception of the ease of use of online teaching affects teachers’ continued usage of online education. Although they did not explore the relationships between perceived usefulness and teacher satisfaction, their findings expressed teachers’ attitudes to a certain extent.

Some interesting findings were recorded when analyzing the relationship between expectation validation, perceived usefulness, and satisfaction. The regression model analysis showed that the hypothesis “Confirmation of teaching goal achievement and online classroom interaction have positive effects on perceived usefulness” was supported. However, hypothesis 10 is not supported. This result is contrary to the existing research on teaching quality (Greimel-Fuhrmann and Geyer, 2003; Song et al., 2016). Generally, student feedback is a vital source for teachers to evaluate their teaching performance, which thereby affects their emotions. Teachers will be confident in their teaching performance when students are active in the classroom (Shoepe et al., 2020). One possible explanation is that a public health emergency could cause schools and teachers to be unprepared when faced with online instruction. While teachers use enormous energy to complete online teaching tasks according to the requirements and regulations, they may still face challenges in paying attention to student feedback. At the same time, online teaching has inherent shortcomings in the perception of students’ attitude feedback. For example, it is difficult for teachers to observe students’ expressions, movements, and other feedback behaviors, which may be possible in face-to-face teaching. Perhaps when they first implemented online teaching in 2020, teachers paid more attention to whether students were satisfied with the quality of their online education. Despite the limitations of attitude feedback in online teaching, teachers have implemented unprepared online teaching many times within three years, and they have become accustomed to the problems, such as low attitude feedback from students. This situation deserves our vigilance. Some objective factors make teachers no longer pay attention to students’ feelings when engaging in online teaching, which deviates teachers’ grasp of students’ learning status and thereby affects the quality of online teaching.

In addition, the reduction of social presence due to space–time barriers and the difficulty of teacher-student interaction has been proved by many studies to be a significant problem affecting online teaching (Gopal et al., 2021). Our results confirm that teachers’ expectations of effective teacher-student interaction significantly impact teachers’ perception of online teaching and satisfaction. This shows that teachers should pay more attention to online teaching design and develop more classroom interaction activities. Teachers should provide more opportunities for interacting with students. They should develop a strategy to combat loneliness and helplessness to improve online teaching efficiency.

Our results also reveal the relational analysis of teachers’ computer self-efficacy on perceived ease of use and satisfaction. Generally, the higher the user’s computer self-efficacy level, the more likely they feel comfortable with the new e-learning method (Katsarou, 2021). However, our results demonstrated that teachers’ computer self-efficacy did not significantly impact their ease of use in online teaching. Teo et al. (2018) also have found that teachers’ computer self-efficacy cannot support ease of use. They explained that even if the teachers were competent enough to accomplish the teaching task through technology, they could still perceive such an effort as laborious. Combined with the characteristics of online teaching during the pandemic, even if teachers are confident in using online technology, they may still perceive online teaching as challenging to implement. Nevertheless, the relationship between teachers’ computer self-efficacy and satisfaction shows that as teachers become more skilled in online teaching, they can solve more issues they meet during teaching, which may enhance their satisfaction with online education. Therefore, more training is vital for teachers to use online teaching platforms and tools.

### 6.2. Theoretical and practical implications

This study contributes to the research on teachers’ satisfaction and performance in the context of online teaching in several aspects. First, our study contributes to the online teaching literature and provides opinions regarding how teachers’ online teaching performance can be improved. Given that little empirical research has been previously conducted to explain the relationship between teachers’ online teaching satisfaction and performance, we explore the factors influencing the satisfaction and performance of teachers for online teaching behavior by proposing an integrated research model (Panisoara et al., 2020). Our results empirically confirmed that teachers’ satisfaction, perceived usefulness, and perceived ease of use of online teaching significantly influence their online teaching performance, thereby providing a framework for future studies to examine the precursors of teachers’ online teaching performance from a usage intention perspective (Sulistiyani and Nugroho, 2022). Meanwhile, the findings reveal that perceived ease of use significantly impacts teacher performance more than perceived usefulness, confirming the pivotal effects of teachers’ familiarity with online teaching technology on online teaching quality. The results also reveal that the relationship between CSE and perceived ease of use is insignificant. Thus, our study provides a possible direction for further research to investigate whether other factors (e.g., quality factors) have predictable effects on teachers’ perceived ease of use of online teaching.

Second, while researchers have acknowledged the influence of teachers’ perceived usefulness and ease of use of online teaching on their satisfaction, few of them have discussed such a relationship exists in an emergency like the one that occurred with COVID-19. To address this research gap, our study applied TAM, ECM, and CSE models to explore the predictors of teachers’ satisfaction with online teaching during COVID-19. The results underscore the critical role of perceived ease of use in achieving teacher satisfaction. This finding suggests that a friendly and easy-of-use online teaching platform may be the precondition for teachers to participate in online teaching. Given our work under the COVID-19 context, with many teachers teaching online without sufficient preparation, our findings may enlighten the research on the large-scale application of online teaching.

Third, this study broadens the application of TAM. Our work is the first research to utilize TAM to explore teachers’ online teaching performance as far as we know. Quite a bit of research on usage intention adopted this model to examine teachers’ and students’ satisfaction and usage intention on classroom IT applications; therefore, it is a practical theory to explain an individual’s behavioral intention. We adopted TAM to explore teachers’ online teaching performance can be a valuable theoretical foundation based on empirical results. Given that our research model has been proven to be strong explanatory power of teachers’ online teaching satisfaction and performance during COVID-19, we may confirm its applicability in the educational context. This model provides an alternative theoretical framework for future research on teachers’ online teaching performance. Further studies incorporating more predictors and extending to the typical types of online teaching may reveal more results on the development of teachers’ online teaching performance.

Our study also provides school administrators and technology providers with practical implications for improving online teaching quality.

First, the results confirm the predictable effects of teachers’ online teaching satisfaction on teachers’ performance. In particular, the findings verify that perceived ease of use exerted a more substantial impact on teachers’ online teaching performance than other variables, which underscored the dominant role of technical convenience (e.g., friendly technical platform, adequate specialized training, timely technical support) in the formation of teachers’ performance. Therefore, school administrators should organize training on online teaching applications, operation guides, and teaching models to improve teachers’ familiarity with online teaching platforms and tools and improve teachers’ online teaching capabilities. Meanwhile, to reduce the difficulty of using the online teaching platform and improve teachers’ usage satisfaction, technology providers are expected to implement more measures to provide timelier response mechanisms in the event of online teaching problems, more concise interactive interfaces, and more straightforward operation procedures.

Second, we found that confirmation of teaching goal achievement and online classroom interaction significantly affect teachers’ perceived usefulness of online teaching. This finding is consistent with previous research on teacher satisfaction with online education (Hampton et al., 2020; Huang et al., 2022). It suggests that school administrators should assist teachers in developing teaching plans calendars before implementing online teaching and clarifying the specific goals of each lesson (Sims and Baker, 2021). Moreover, they can organize training for highly interactive teaching tools to help teachers acquire interactive online teaching skills.

Third, as the effects of confirmation of student satisfaction cannot influence teachers’ perception and satisfaction with online teaching usefulness, the unexpected result should attract the attention of researchers and practitioners. As teachers seemed not to care about students’ feedback regarding the teaching quality and ignored the rigorous and systematic design of remote teaching and learning, we wonder if they just took online teaching as a temporary alternative to cope with the teaching task during the epidemic. School administrators should take decisive measures, such as strengthening the monitoring of teachers’ online teaching process, and using more flexible methods to obtain feedback on teachers’ online teaching, to guarantee the quality of online education.

## 7. Conclusion

This study uses the integrated model of TAM, ECM, and CSE to investigate factors influencing Chinese university teachers’ satisfaction and online teaching performance. Since few studies systematically examine teacher satisfaction and performance, we explored this area from an integrative perspective. The results show that teachers’ satisfaction with online teaching has a significant impact on teachers’ online teaching performance, which provides a conditional basis for teaching reform and for enhancing the quality of online education and teacher satisfaction. In perceived ease of use and perceived usefulness, we have found that teachers are more aware of the satisfaction in ease of use. In addition, teachers’ perception of usefulness and satisfaction with online teaching is mainly on whether online teaching can effectively achieve teaching goals and whether effective classroom interaction is available during the teaching process. However, students’ attitudes and their feedback on courses and teachers did not affect teachers’ perspectives on the usefulness of online teaching. Possible constraints in the online teaching task and computer self-efficacy were predictors of teachers’ online teaching satisfaction but did not affect their perception of the ease of use of online teaching. Our findings will enrich the literature on teachers’ online teaching performance and increase our understanding of the antecedents of teachers’ online teaching satisfaction. Our findings will also assist school administrators and technology providers to improve their teaching support and technology tool systems.

## 8. Limitations

Two limitations found in our study are as follows: First, the sampled universities are all from developed cities, such as Shanghai. Therefore, the quality of network equipment and online teaching platform tools is best guaranteed in these cities. We are uncertain whether network equipment quality factors contribute to teacher satisfaction. Second, we did not specifically consider the impact of different knowledge types on teachers’ online teaching satisfaction and performance. Perhaps online teaching in theoretical courses is more accessible than online teaching in practical knowledge. However, we have not yet verified this with empirical data. Future research can improve this by analyzing the different courses in the same subject area.

## Data availability statement

The raw data supporting the conclusions of this article will be made available by the authors, without undue reservation.

## Author contributions

WD, LW, and RL discussed the research ideas, research methods, and framework of the article. Specifically, WD is mainly responsible for conceptualization, literature review, and discussion. JZ is responsible for discussion. LW and RL are responsible for data collection, data analysis, and the conclusion section. WD and RL are responsible for initial draft preparation. WD, LW, and JZ are responsible for revision and editing. All authors have read and agreed to the published version of the manuscript.

## Funding

The Xie You Bai Design Science Research Foundation, grant number XYB-DS-202002; Undergraduate Educational Reform Research Project of Jiangnan University, grant number JG2021139.

## Conflict of interest

The authors declare that the research was conducted in the absence of any commercial or financial relationships that could be construed as a potential conflict of interest.

## Publisher’s note

All claims expressed in this article are solely those of the authors and do not necessarily represent those of their affiliated organizations, or those of the publisher, the editors and the reviewers. Any product that may be evaluated in this article, or claim that may be made by its manufacturer, is not guaranteed or endorsed by the publisher.
